# One-carbon pathway metabolites are altered in the plasma of subjects with Down syndrome: Relation to chromosomal dosage

**DOI:** 10.3389/fmed.2022.1006891

**Published:** 2022-12-01

**Authors:** Beatrice Vione, Giuseppe Ramacieri, Giacomo Zavaroni, Angela Piano, Giorgia La Rocca, Maria Caracausi, Lorenza Vitale, Allison Piovesan, Caterina Gori, Gian Luca Pirazzoli, Pierluigi Strippoli, Guido Cocchi, Luigi Corvaglia, Chiara Locatelli, Maria Chiara Pelleri, Francesca Antonaros

**Affiliations:** ^1^Department of Experimental, Diagnostic and Specialty Medicine (DIMES), Unit of Histology, Embryology and Applied Biology, University of Bologna, Bologna, Italy; ^2^Department of Medical and Surgical Sciences (DIMEC), University of Bologna, Bologna, Italy; ^3^Medical Department, Maggiore Hospital, Bologna, Italy; ^4^Neonatology Unit, IRCCS Sant’Orsola-Malpighi University Hospital, Bologna, Italy

**Keywords:** trisomy 21, Down syndrome, one-carbon pathway, folates, chromosomal dosage

## Abstract

**Introduction:**

Down syndrome (DS) is the most common chromosomal disorder and it is caused by trisomy of chromosome 21 (Hsa21). Subjects with DS show a large heterogeneity of phenotypes and the most constant clinical features present are typical facies and intellectual disability (ID). Several studies demonstrated that trisomy 21 causes an alteration in the metabolic profile, involving among all the one-carbon cycle.

**Methods:**

We performed enzyme-linked immunosorbent assays (ELISAs) to identify the concentration of 5 different intermediates of the one-carbon cycle in plasma samples obtained from a total of 164 subjects with DS compared to 54 euploid subjects. We investigated: tetrahydrofolate (THF; DS *n* = 108, control *n* = 41), 5-methyltetrahydrofolate (5-methyl-THF; DS *n* = 140, control *n* = 34), 5-formyltetrahydrofolate (5-formyl-THF; DS *n* = 80, control *n* = 21), S-adenosyl-homocysteine (SAH; DS *n* = 94, control *n* = 20) and S-adenosyl-methionine (SAM; DS *n* = 24, control *n* = 15).

**Results:**

Results highlight specific alterations of THF with a median concentration ratio DS/control of 2:3, a decrease of a necessary molecule perfectly consistent with a chromosomal dosage effect. Moreover, SAM and SAH show a ratio DS/control of 1.82:1 and 3.6:1, respectively.

**Discussion:**

The relevance of these results for the biology of intelligence and its impairment in trisomy 21 is discussed, leading to the final proposal of 5-methyl-THF as the best candidate for a clinical trial aimed at restoring the dysregulation of one-carbon cycle in trisomy 21, possibly improving cognitive skills of subjects with DS.

## Introduction

Down syndrome (DS) or trisomy 21 (T21) is the most frequent aneuploidy occurring in human live births (OMIM 190685) and the most common genetic cause of intellectual disability (ID) (1–3). Its association with the presence of an extra copy of a chromosome 21 (Hsa21) was discovered only in 1959 by Jérôme Lejeune and coll (4).

Lejeune was also the first who presented a general analysis of the pathogenesis of metabolic diseases determining ID, hypothesizing that DS could be also considered a metabolic disease (5). Following Lejeune, we conducted a nuclear magnetic resonance (NMR) analysis of the metabolome in plasma and urine samples that showed a clear difference between trisomic and euploid subjects (6), confirmed in a more recent study in plasma samples of a greater cohort (7). The univariate analysis showed a significant alteration of some metabolites. Most of the altered concentrations reflected the 3:2 gene dosage model, suggesting effects caused by the presence of three copies of Hsa21 on metabolites involved in central metabolic processes such as fumarate and succinate in the Krebs cycle, pyruvate and lactate in glycolysis, and formate, an end product of one-carbon metabolism in the mitochondrion. These alterations, as suggested by other studies (8, 9), may be an expression of a shift from an aerobial pathway to an anaerobic one as well as an expression of a hypoxic state of the cell that causes higher mitochondrion stress. This stress is not adequately borne by DS cells due to alterations in central carbon metabolism (10).

**FIGURE 1:**
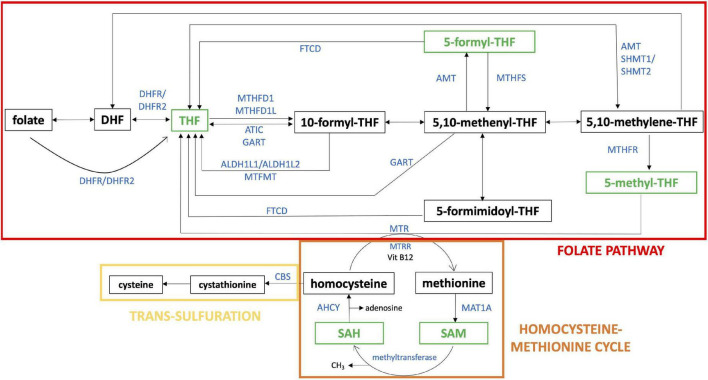
One-carbon pathway. The figure shows a schematic representation of folate pathway (in red), homocysteine-methionine cycle (in orange) and trans-sulfulration pathway (in yellow), that are part of one-carbon pathway. The metabolites analyzed in our study are reported in green and the enzymes directly involved in their production or transformation are indicated with the official symbol reported in Gene NCBI (https://www.ncbi.nlm.nih.gov/gene). Alternative forms of enzyme are shown together separated by “/”. The schematic representation was realized starting from “One carbon pool by folate - Homo sapiens” of KEGG pathway database (https://www.genome.jp/kegg/pathway.html).

One-carbon metabolism (Figure 1) is the process by which one-carbon groups at different oxidation states are used in a set of interconnected biochemical pathways driven by folate and homocysteine-methionine cycles, involved in DNA synthesis through purine and thymidylate generation, amino acid homeostasis, antioxidant generation, and epigenetic regulation (11). Remethylation of homocysteine to methionine allows several transmethylation reactions. Folate metabolism plays at least two separate roles. It is critical for one-carbon metabolism. Moreover, reactions of folate metabolism play a role in the catabolism of choline and at least three amino acids: histidine, serine and glycine (12). These biochemical processes, in turn, support critical cellular functions such as cell proliferation, mitochondrial respiration and epigenetic regulation (13).

In his 1979 pivotal study Lejeune, comparing DS with several metabolic diseases, hypothesized that a disturbance of the one-carbon cycle could occur in DS. He thought that trisomic cells were intoxicated by an excess of gene products, which was caused by the presence of the additional Hsa21 (14). He investigated the genes located on Hsa21, and he found that several enzymes involved in the one-carbon metabolism were encoded by genes located on this chromosome, in particular: cystathionine β-synthase (*CBS*) and phosphoribosylglycinamide formyltransferase, phosphoribosylglycinamide synthetase, phosphoribosylaminoimidazole synthetase (the three subunits of GART enzyme). Moreover, the gene for the main transporter of folate, solute carrier family 19 member 1 gene (*SLC19A1*), is also located on Hsa21.

Further studies performed by Lejeune and coll. demonstrated that methotrexate (MTX) was twice as toxic in T21 lymphocytes as compared to control cells (15), in agreement with the observation of Peeters and coll. of an increased toxicity of MTX in the leukemia therapy in children with DS compared to euploid subjects (16). MTX is a cytotoxic drug used as a chemotherapy agent and immune system suppressant (17, 18). MTX is a folate analog which inhibits the activity of the dihydrofolate reductase (DHFR) enzyme involved in the one-carbon metabolic cycle (15).

In 2019 we evaluated the rescue effect on MTX toxicity mediated by folate and some of its derivatives in primary human fibroblast cell lines obtained from euploid and T21 donors (19). This study shows that 5-methyltetrahydrofolate (5-methyl-THF) and 5-formyltetrahydrofolate (5-formyl-THF or folinic acid), but not tetrahydrofolate (THF), treatments have significant protective effects on both euploid and T21 cells during MTX treatment.

In a recent survey, erythrocyte folate concentrations in children with DS were compared with a cohort of subjects with juvenile arthritis as euploid control, in order to understand if the MTX treatment toxicity in children with DS is related to altered erythrocyte folate concentrations. They reported a lower concentration level of total folate, 5-methyl-THF and 5,10-methenyl-THF in the erythrocytes of subjects with DS. These reductions in erythrocyte folates were also associated with a decrease in short-chain folate polyglutamation 5-methyl-THFGlu3-6 and a corresponding increase in longer chain 5-methyl-THFGlu7-10 (20). These data suggest that in trisomic cells there is a biological adaptation to folate deficiency by an increased level of long-chain folate polyglutamation; indeed, increased expression of folylpolyglutamate synthetase (*FPGS*) gene was already reported *in vitro* and in animal models (20–22).

In subjects with DS, one-carbon metabolism was considered to be imbalanced by several authors (5, 20, 23–25). In particular, subjects with DS commonly present lower blood levels of vitamin B12 (or cobalamin) and folic acid with increasing age and lower erythrocyte folates and homocysteine (Hcy) compared to healthy controls (20, 25–27).

THF levels in DS subjects’ plasma was never investigated. Our study is aimed to systematically analyze by sensitive enzyme-linked immunosorbent assays (ELISAs) several metabolites involved in one-carbon metabolism both in subjects with DS and their siblings to understand if there are differences likely to be associated with the presence of a third copy of Hsa21. THF, 5-methyl-THF, 5-formyl-THF, S-adenosyl-homocysteine (SAH) and S-adenosyl-methionine (SAM) were analyzed in plasma samples obtained from a total of 164 subjects with DS and 54 euploid subjects as control along with serum folic acid, serum vitamin B12 and serum Hcy in the DS group.

## Materials and methods

### Ethics statement

The Independent Ethics Committee of the Hospital - University of Bologna Policlinico S. Orsola-Malpighi Italy has granted the ethical approval for this study (number: 39/2013/U/Tess). We obtained an informed written consent from all participants to collect urine and blood samples and clinical data. Concerning minors, the consent was collected from his/her parents. All procedures were carried out in accordance with the Ethical Principles for Medical Research Involving Human Subjects of the Helsinki Declaration.

### Case selection

A total of 218 subjects were selected for this study, including 164 subjects with DS and 54 subjects as control, selected among siblings of subjects with DS and without evidence of abnormal karyotype. The study has been proposed to all subjects consecutively admitted to the Day Hospital of the Neonatology Unit, IRCCS Sant’Orsola-Malpighi Polyclinic, Bologna, Italy, in the context of routine follow up provided for DS.

For this work, we considered in the DS group subjects with diagnosis of DS with homogeneous or mosaic T21, availability of an adequate amount of plasma to perform at least one ELISA assay and a similar mean age as close as possible to control group. Concerning control group, we considered subjects with an adequate amount of plasma to perform at least one ELISA assay and a similar mean age as close as possible to DS group. The blood samples selected were treated within two hours of collection and plasma samples contaminated by residual erythrocytes after the treatment were excluded.

DS group consists of 95 males (M) and 69 females (F) with a mean age of 11.55 years old (standard deviation, SD = 6.69) and an age range from 3.1 to 37.9; control group consists of 30 M and 24 F with a mean age of 14.53 years old (standard deviation, SD = 6.64) and an age range from 3.3 to 31.5. Concerning the control group, 29 subjects were siblings of 23 subjects in the DS group.

For every collected sample, parents filled out a form with information about the current fasting state, last meal, consumed medications (see Supplementary Data Sheet 1).

### Blood sample preparation

For DS group, two blood sample aliquots were collected in EDTA-coated blood collection tubes in the Neonatology unit.

The first aliquot was sent to “Laboratorio unico Metropolitano” (LUM) of Maggiore Hospital (Bologna, Italy) for routine blood analyses of the DS group including folic acid and vitamin B12. The concentration levels in serum were obtained by chemiluminescent immunoassay by Beckman Coulter Immunoassay Systems (Beckman Coulter, respectively REF A98032 and REF 33000).

The second aliquot was kept at room temperature and treated within two hours from blood draw for plasma isolation.

For control group, one blood sample aliquot were collected in EDTA-coated blood collection tubes in the Neonatology unit. The second aliquot was kept at room temperature and treated within two hours from blood draw for plasma isolation.

Plasma isolation for DS and control aliquots was performed as following. Sample was transferred to a new tube and centrifuged at 1,250 g for 10 min to separate corpuscular fraction from plasma. The plasma was isolated and centrifuged for a second time at 800 g for 30 min to eliminate residual debris. The supernatant was collected and transferred into new tubes avoiding contact with pellets or the bottom of the tube, divided in aliquots and rapidly stored in a −80°C freezer.

Every delayed treatment of the sample following its transfer to the university laboratory (> 2 h) was recorded, as one of the exclusion criteria from the analysis was sample treatment after two hours from the draw. The other one was evident contamination of plasma by hemoglobin due to hemolysis after the treatment. All procedures were conducted carefully to avoid contamination during the different steps and any anomalies like different plasma color or precipitates after centrifugation were noted and considered in further analysis.

One or more plasma aliquots were used for ELISA assays. For DS group only, one plasma aliquot was sent to LUM for the detection of Hcy plasma level by an automated chemistry analyzer AU 400 Beckman Coulter (Beckman Coulter, REF FHRWAU/FHRWR100/200/1000).

Concerning concentration values of folic acid, vitamin B12 and Hcy of DS group, 25% of data that are present in our Supplementary Data Sheet 1 can be found also in the Supplementary Data Sheet 1 of our previously published work in (28).

### Enzyme-linked immunosorbent assay

We evaluated the quantitative measurement of THF, 5-methyl-THF, 5-formyl-THF and SAH plasma concentrations using specific ELISA kits manufactured by MyBioSource (San Diego, California, USA) and SAM plasma concentration using a specific ELISA kit manufactured by Biovision (Milpitas, California, USA).

“General Tetrahydrofolic Acid (THFA) ELISA assay,” “Human 5-methyltetrahydrofolate ELISA assay,” and “Enzyme-linked Immunosorbent assay for 5-formyl-THF (folinic acid)” MyBioSource kits are based on a competitive inhibition enzyme technique.

Regarding THF, specifications were: detection range, 1.23-100 ng/ml; sensitivity, 0.57 ng/mL; intra-assay precision, Coefficient of Variation (CV) < 10%; inter-assay precision, CV < 12%; no significant cross-reactivity or interference between THFA and analogs was observed.

Regarding 5-methyl-THF, assay specifications were: detection range, 0.156-10 ng/ml; sensitivity, 0.094 ng/ml; intra-assay precision, CV < 8%; inter-assay precision, CV < 10%; no significant cross-reactivity or interference between 5-methyl-THF and analogs was observed.

Regarding 5-formyl-THF, assay specifications were: detection range, 117.2-30,000 pg/ml; sensitivity, <41.5 pg/ml; intra-assay precision, CV < 10%; inter-assay precision, CV < 12%; no significant cross-reactivity or interference between Folinic Acid (FA) and analogs was observed.

MyBioSource “Human S-Adenosyl-Homocysteine (SAH) ELISA assay” and Biovision “S-Adenosylmethionine (SAM) ELISA” kits employ a double antibody sandwich technique.

Regarding SAH, assay specifications were: detection range, 20 ng/ml-0.312 ng/ml; sensitivity, up to 0.06 ng/ml; intra-assay precision, CV **≤** 8%; inter-assay precision, CV **≤** 12%; no cross-reaction with other factors.

Regarding SAM, assay specifications were: detection range, 0.39-25 mcg/ml; sensitivity, < 0.234 mcg/ml; intra-assay precision, CV < 8%; inter-assay precision, CV < 10%; no significant cross-reactivity or interference between SAM and analogs was observed.

Ninety-six well plates were used for all the assays and all standard and plasma samples were tested in duplicate. Due to preliminary studies, plasma samples used for the measurement of 5-methyl-THF were diluted 1:10 using Sample Dilution Buffer supplied by the kit and plasma samples used for the measurement of SAH were diluted 1:5 in Plates 1, 2, 3, and 4, and 1:3 in Plates 4 and 5 to avoid excessive dilution of the metabolite, using Sample Diluent supplied by the kit. The standard samples were provided by the kits and were reconstituted and serially diluted as suggested.

In order to perform the assays, the manufacturer instructions of each kit were followed, and the final spectrophotometric reading was carried out by microplate reader (Perkin Elmer Wallac 1,420 Victor 2 Multi-Label) set at a wavelength of 450 nm.

In order to create the standard curve for each ELISA assay, standard O.D. mean values were plotted on the *x*-axis and the known standard concentration values were plotted on the y-axis using Microsoft Excel and following manufacturer instructions. The transformation of standard concentration values in their logarithm (Log_10_) is required to build the standard curves in THF and 5-formyl-THF ELISA assays. The subtraction of the background O.D. mean value (“standard 8”) from the O.D. mean values of the other standards and plasma samples is required in SAH ELISA assay. The transformation of standard O.D. mean values in their inverse (1/O.D. mean) is required to build the standard curves in the case of the SAM assay.

The polynomial trend lines of each plot were created using Excel, and the resulting polynomial equations (*y* = a + bx + cx^1^) were used to determine metabolite concentrations of plasma samples using interpolation.

### Statistical analyses

Statistical analyses were carried out with SPSS Statistics software (IBM, Version 25 for Mac OS X) and were performed using the data available in Supplementary Data Sheet 1. For all results, a *p* < 0.05 was considered statistically significant. An *r* < 0.4 was considered as weakly correlated, 0.4 < *r* < 0.7 as moderately correlated and *r* > 0.7 as strongly correlated.

We used SPSS Statistics to perform a first descriptive analysis in order to obtain a general view of the data included in Supplementary Data Sheet 1 and to highlight the presence of strong outliers in concentration level distribution. SPSS considers data as an outlier if it is outside the following ranges: above the 3rd quartile + 1.5 interquartile range or below the 1st quartile −1.5 interquartile range and indicates it with an asterisk in the graph.

To check if our data followed a normal distribution we used Kolmogorov-Smirnov test using Social Science Statistic software online^2^

For each metabolite (THF, 5-methyl-THF, SAH, and SAM) we performed an unpaired student t-test between DS and control group using the “Graph Pad” *t*-test calculator online^2^, while for 5-formyl-THF levels, that resulted to be distributed in a way significantly different from a normal distribution, we performed Mann-Whitney test online^3^.

**FIGURE 2:**
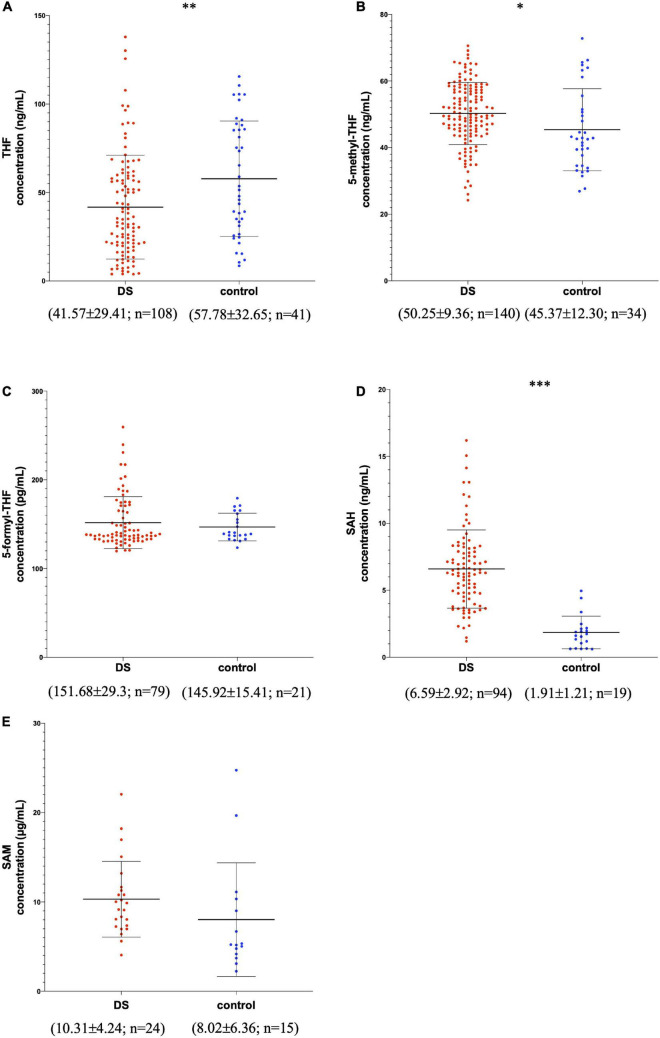
Metabolite concentrations in subjects with DS and normal control subjects. The graphs report metabolite plasma levels of each subject in the study. On the *x*-axis there is the subdivision of subjects in DS and control groups. Subjects with DS are represented like red dots and normal control subjects are represented like blue dots. On the y-axis the concentration of the metabolite in μg/ml, ng/ml or pg/ml is reported. The asterisks above the graph indicate the level of statistical significance (*p < 0.05; ^**^p < 0.005; ^***^p < 0.0005). The middle black lines indicate the mean concentration values for each group and the external black lines indicate standard deviation (SD) values. The mean concentration, SD values and the number of subjects (n) are reported below each graph for DS and control groups. **(A)** shows THF concentrations; **(B)** shows 5-methyl-THF concentrations; **(C)** shows 5-formyl-THF concentrations excluding strong outliers; **(D)** shows SAH concentrations excluding strong outliers; **(E)** shows SAM concentrations. The graphs were created with GraphPad Prism software v.6.0 (San Diego, CA).

The graphic reports of each subject’s metabolite plasma levels in the study were created with GraphPad Prism software v.6.0 (San Diego, CA) (Figure 2 and Supplementary Figure 1).

In SPSS Statistics we used linear correlation to search for correlation between age and molecule levels and unpaired *t*-test to verify whether sex and fasting/non-fasting state might affect the main results.

SPSS Statistics was used to perform a linear correlation between the level of each molecule and the levels of all the other molecules. Partial correlation analyses checked for the effect of chronological age were used to investigate associations between the level of the involved molecules and other molecules.

**FIGURE 3:**
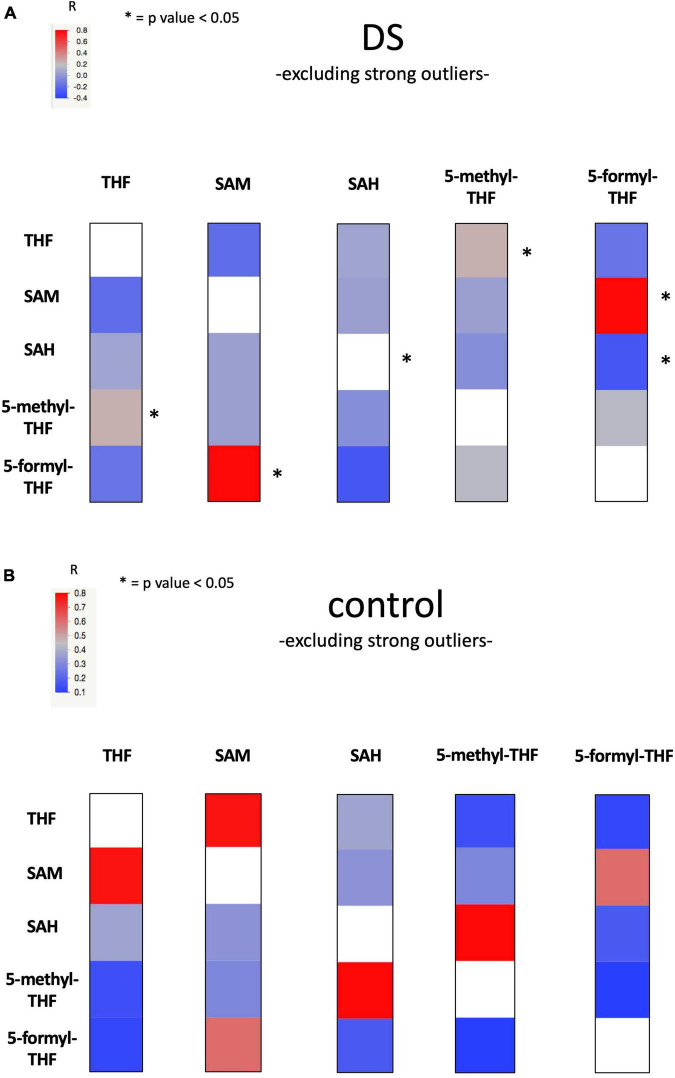
Heat Map figure of bivariate correlation between levels of each metabolite and levels of all the other metabolites excluding strong outliers. **(A)** presents bivariate correlations in DS group and **(B)** presents bivariate correlation in the control group (for complete data see Supplementary Table 11). At the top left of the figures the color code bar for Pearson correlation coefficient (r). Statistically significant correlations were marked with an “*”.

The Heat Map figures (Figure 3 and Supplementary Figure 2) representing metabolite correlations (THF, 5-methyl-THF, 5-formyl-THF, SAH and SAM) were generated using JMP Pro software (Version 14 of the SAS System for Mac OS X, SAS Institute Inc., Cary, NC, USA).

The exact procedures used to perform all statistical tests are detailed in Supplementary Data Sheet 1.

## Results

### Enzyme-linked immunosorbent assay

We selected 164 subjects with DS and 54 euploid subjects. Concerning subjects with DS, only one has T21 mosaicism at 94%. In the DS group we obtained the concentration values of THF in 108 subjects, of 5-methyl-THF in 140 subjects, of 5-formyl-THF in 80 subjects, of SAH in 94 subjects and of SAM in 24 subjects. In the control group we obtained the concentration values of THF in 41 subjects, of 5-methyl-THF in 34 subjects, of 5-formyl-THF in 21 subjects, of SAH in 20 subjects and of SAM in 15 subjects (see Supplementary Data Sheet 1). It was not possible to obtain the same number of measurements of metabolite levels in all the selected subjects for several reasons: the amount of plasma was not sufficient to perform ELISA assay for each metabolite, the absorbance (O.D., optical density) mean values of plasma samples were out of the range, or there was a technical problem during the assay. Moreover, it was not possible to have a larger group of SAM concentration results due to technical problems with some of the ELISA kits purchased.

Concerning the O.D. mean values, those that were higher or lower than the O.D. mean values of standard sample ranges were not taken into consideration (they are indicated in red in Supplementary Tables 1, 3–5). In total we did not consider the concentration values of 9 DS plasma samples in THF ELISA assays, 55 DS and 8 control plasma samples in 5-formyl-THF assays, 18 DS and 14 control plasma samples in SAH ELISA assays because their detection rate was lower than the minimum of the standard range and 1 plasma sample in SAM ELISA assay because its concentration was higher than the maximum of the standard range.

Considering the analysis of 5-methyl-THF and SAM, there was no significant loss between DS and control samples. In the THF analysis, the DS samples had a loss of 8% while the control samples had no loss. The loss rate was higher with the analysis of 5-formyl-THF and SAH, where there was a loss of 41% in DS samples and 28% in control samples with 5-formyl-THF analysis and a loss of 16% in DS samples and 41% in control samples with SAH analysis. However, the decrease in sample size due to its loss could only be a problem for the non-statistically significant result for 5-formyl-THF, although, assuming a medium/large effect size, the *post hoc* analysis achieves a power of 0.80.

In order to assess the intra-replicate variability of the assays in duplicate, the Coefficient of Variation of the O.D. values expressed as percentage (%CV) was reported for each sample in Supplementary Tables 1–5. The %CV was calculated dividing the standard deviation of the two measurements for the mean of the two measurements and multiplying for 100. The mean of the %CV values also named Intra-Assay CV was 9.55% for THF assays, 6.77% for 5-methyl-THF assays, 6.92% for 5-formyl-THF assays, 10.95% for SAH assays and 8.40% for SAM assay. While the mean of the %CV values was lower than 11% for all five metabolites, individual values could exceed 15% in a part of the measured sample. In particular, out of a total of 189 samples assayed there were 40 samples that ranged between 15.49% and 53.23% of %CV in THF assays; out of a total of 222 samples assayed, there were 17 samples that ranged between 15.03% and 31.15% in 5-methyl-THF assays; out of a total of 143 samples assayed, there were 15 samples that ranged between 15.44% and 36.29% in 5-formyl-THF assays; out of a total of 162 samples assayed, there were 46 samples that ranged between 15.10% and 46.36% in SAH assays; out of a total of 47 samples assayed, there were 8 samples that ranged between 15.12 and 72.55% in SAM assay.

To obtain a standard curve in order to quantify the plasma metabolites, we checked the determination coefficient R^2^ values for both linear and polynomial of grade 2 (quadratic) curves. The best fit for all the plates was always obtained using a quadratic equation (for one out of 24 plates, namely Plate 4 of SAH analysis, R^2^ values of linear and quadratic equations were identical, data not shown). R^2^ of the standard curves determined in this way ranged between 0.9697 and 0.9881 for THF (see Supplementary Table 1), between 0.922 and 0.9707 for 5-methyl-THF (see Supplementary Table 2), between 0.9048 and 0.9433 for 5-formyl-THF (see Supplementary Table 3), between 0.9998 and 0.9956 for SAH (see Supplementary Table 4) and 0.9996 for SAM (see Supplementary Table 5).

In order to look for correlation between level of metabolites analyzed by ELISA tests and levels of other available metabolites, for the DS group we collected the concentration values in serum of folic acid in 143 subjects and of vitamin B12 in 155 subjects and of plasma Hcy in 42 subjects [see Supplementary Data Sheet 1, 25% of data that are present in our Supplementary Data Sheet 1 can be found also in the Supplementary Data Sheet 1 of our previously published work in 2021 (28)].

### Statistical analyses

Overall, 3 strong outliers were identified among 5-formyl-THF and vitamin B12 concentration values of the DS group and SAH concentration values of control group that are reported in red in Supplementary Data Sheet 1.

The main results of descriptive analyses of THF, 5-methyl-THF, 5-formyl-THF, SAH and SAM without strong outliers are shown in Table 1. The detailed results of descriptive analysis with and without strong outliers are shown in Supplementary Table 6.

**TABLE 1:** Main results of descriptive analysis of DS and control group excluding strong outliers.

	DS (*n* = 164)					control (*n* = 54)				
	THF (ng/ml)	5-methyl-THF (ng/ml)	5-formyl-THF (pg/ml)	SAH (ng/ml)	SAM (μg/ml)	THF (ng/ml)	5-methyl-THF (ng/ml)	5-formyl-THF (pg/ml)	SAH (ng/ml)	SAM (μg/ml)
Valid n =	108	140	79	94	24	41	34	21	19	15
Missing n =	56	24	85	70	140	13	20	33	35	39
Mean	41.574	50.247	151.675	6.588	10.308	57.783	45.369	145.917	1.911	8.022
Median	33.955	49.766	137.955	6.298	9.514	51.471	42.744	139.144	1.736	5.222
SD	29.414	9.358	29.300	2.923	4.237	32.645	12.301	15.413	1.213	6.362

The values of subjects with Down syndrome (DS) are reported on the left of the table and the values of normal control subjects (control) are reported on the right of the table. For each metabolite the number (n) of plasma samples valid or missing are reported. Also the mean, median and standard deviation (SD) values are shown. The detailed results of descriptive analysis with and without strong outliers are shown in Supplementary Table 6.

The results of unpaired student t-test between DS and control concentration values of each metabolite showed that the difference between DS and control concentrations is statistically significant for THF (*p*-value = 0.0041), 5-methyl-THF (*p*-value = 0.015) and SAH (*p*-value = 0.0001) when the strong outliers are excluded (see Table 2) or included (see Supplementary Table 7) in the analyses. The results of Mann-Whitney test between DS and control 5-formyl-THF concentration values is not statistically significant when the strong outliers are included (*p*-value = 0.88076) or excluded (*p*-value = 0.81034) (see Supplementary Table 7).

**TABLE 2:** Difference of metabolite concentration between DS and control groups.

	THF	5-methyl-THF	5-formyl-THF	SAH	SAM
Subjects	DS (*n* = 108)	DS (*n* = 140)	DS (*n* = 79)	DS (*n* = 94)	DS (*n* = 24)
	Control (*n* = 41)	Control (*n* = 34)	Control (*n* = 21)	Control (*n* = 19)	Control (*n* = 15)
Mean ratio DS/Control	0.72	1.11	1.04	3.45	1.28
Median ratio DS/Control	0.66	1.16	0.99	3.63	1.82
p-value	0.0041*	0.0115*	0.8103	0.0001*	0.1853
t-value	2.9137	2.5537	801^a^	6.8373	1.3499

Strong outliers are excluded. Mean and median concentration values are given in Table 1. Ratio of mean and median values between DS and control groups are given. Statistical test was t-student except for 5-formyl-THF for which Mann-Whitney test was used. Significant *p*-values (<0.05) are marked with an “*”. The detailed results with and without strong outliers are shown in Supplementary Table 7.

^a^*U*-value for Mann-Whitney test.

Figure 2 shows the variation of THF, 5-methyl-THF, 5-formyl-THF, SAH, and SAM concentrations in subjects with DS and normal control subjects excluding strong outliers. In Supplementary Figure 1 the variation of 5-formyl-THF and SAH concentration in subjects with DS and normal control subjects is reported including strong outliers.

In Table 2 the ratio of mean concentration values and the ratio of median concentration values between DS and control groups is reported.

A statistically significant weak correlation (*r* = Pearson correlation coefficient) was found between age and THF concentration levels in control group (*r* = 0.395 and *p*-value = 0.011). The significance was lost in DS group. Whether or not the strong outliers were considered did not change the results (see, respectively Table 3 and Supplementary Table 8B). A statistically significant moderate correlation was found between age and Hcy concentration levels in the DS group (*r* = 0.593 and *p*-value < 0.001) (see Supplementary Tables 8A,B).

**TABLE 3:** Bivariate correlation between age and each concentration level in DS and control groups excluding strong outliers.

	DS					Control				
	THF	5-methyl-THF	5-formyl-THF	SAH	SAM	THF	5-methyl-THF	5-formyl-THF	SAH	SAM
r	0.089	-0.085	0.128	0.152	0.099	0.395	0.271	0.239	0.102	0.115
p-value	0.362	0.318	0.261	0.145	0.644	0.011*	0.121	0.296	0.678	0.682

The table reports for each metabolite the results of the statistical analyses in DS and control groups indicated by Pearson correlation coefficient (r) and two-tailed significance (*p*-value). Significant r (>0.4) and *p*-values (<0.05) are marked with an “*”. The detailed results of bivariate correlation with and without strong outliers are shown in Supplementary Table 8.

The unpaired t-test did not identify differences between males and females concerning the concentration of all the molecules investigated (see Supplementary Table 9).

The unpaired *t*-tests comparing values in fasting or non-fasting state highlighted a statistically significant difference in vitamin B12 levels in the DS group when the strong outliers are both included (*p*-value = 0.004) (see Supplementary Table 10A) or excluded (*p*-value = 0.005) (see Supplementary Table 10B) in the analyses.

The linear correlation analysis identified a statistically significant moderate negative correlation (*r* = −0.628 and *p*-value = 0.029) between SAM and vitamin B12 levels in the non-fasting DS group. A statistically significant strong positive correlation (*r* = 0.81 and *p*-value = 0.003) between SAM and 5-formyl-THF levels was found in DS group. The correlation was not maintained in the control group (see Supplementary Table 11, Figure 3 and Supplementary Figure 2).

## Discussion

Our results highlight plasma level alteration of some intermediates of one-carbon metabolism in a group of subjects with DS compared to a group of euploid subjects as control. We were able to enroll a lower number of control subjects compared to subjects with DS, a common situation (see for example (6, 7, 20, 29) often due to the difficulty in the enrollment of healthy children in a medical setting.

We reported a statistically significant difference of THF (*p*-value = 0.0041), 5-methyl-THF (*p*-value = 0.015) and SAH (*p*-value = 0.0001) plasma levels between DS and control groups (see Table 2). Concerning 5-formyl-THF and SAM, the difference is not statistically significant (respectively *p*-value = 0.8103 and 0.1853) (see Table 2).

THF is the metabolic active form of folates in cells and it serves as one-carbon carrier in most folate-mediated reactions (30). THF is the folate form most interconnected in the folate pathway and it is the product of ten enzymatic reactions as shown in Figure 1. Moreover, Lejeune analyzed the genetic diseases causing biochemical alterations that affect the nervous system and he found out that many biochemical dysregulations had an effect on THF metabolism and its chemical connections (5). Our results suggest that THF plasma concentration is lower than normal in subjects with DS and the median concentration ratio between DS and control groups is 0.66 (see Table 2), that is a 2:3 ratio, strongly suggesting a correlation with the imbalanced original event or the presence of a third Hsa21. It has been repeatedly reported over decades that only a fraction of Hsa21, within 21q22, is strictly associated with the clinical diagnosis of DS (31, 32), and it has recently been delimitated as “highly restricted Down syndrome critical region” (HR-DSCR; (33, 34). Considering that DS phenotype is dominated by the constant presence of ID, we could hypothesize that one-carbon cycle might be a main driver of ID and thus likely to be affected by genetic determinants in the Hsa21 “critical region,” rather than by other Hsa21 genes cited in the Introduction section and located outside it. This hypothesis could be verified by dosing cellular content of different forms of folate following removal by genetic engineering of one copy of HR-DSCR from cultured trisomic cells, and experiments are in progress in this regard in our Laboratory.

Moreover, THF concentration level shows a statistically significant weak correlation with age in the control group (*r* = 0.395 and *p*-value = 0.011) (see Table 3). Pfeiffer and coll. reported that older age is associated with less bioactive folate (THF) and more biologically inactive folate (35). Interestingly, our results report that the correlation between THF and age is lost in the DS group (*r* = 0.089 and *p*-value = 0.362) suggesting that the plasma level alteration of THF in subjects may be a stable consequence of trisomy 21 masking variation with age.

5-methyl-THF is the most abundant folate form in blood circulation and the only form able to cross the blood-brain barrier (36). Our results suggest that 5-methyl-THF plasma concentration is slightly higher than normal in subjects with DS and the median concentration ratio between DS and control groups is 1.16 (see Table 2), that is a 1:1 ratio.

5-formyl-THF is a reserve of one-carbon units in cells, and it does not play a direct biosynthetic role (36). Our results suggest that 5-formyl-THF plasma concentration is equal in subjects with DS compared to normal control subjects and the median concentration ratio between DS and control groups is 0.99 (see Table 2), that is a 1:1 ratio.

SAH is part of homocysteine-methionine cycle in which the universal methyl donor named S-adenosyl-methionine (SAM) is transformed in SAH by methyltransferases (37) (see Figure 1). Our results suggest that SAH plasma concentration is much higher than normal in subjects with DS and the median concentration ratio between DS and control groups is 3.63 (see Table 2).

Even if there is not a statistically significant difference of SAM plasma levels between the DS and control groups due to the distribution of the values (see Table 2), SAM concentration is higher than normal in subjects with DS and the median concentration ratio between DS and control groups is 1.82 (see Table 2).

In a previous work (27), both SAH and SAM were found to be significantly higher in young individuals with DS compared to control subjects of comparable age, and the median SAM/SAH ratio was lower in individuals with DS. We find here that, following data in Table 1, the SAM/SAH mass median ratio is 1.511 in subjects with DS and 3.008 in control subjects, suggesting that SAM/SAH median ratio in subjects with DS is half (exactly 0.50) compared to control subjects. SAM/SAH ratio is a well known indicator of cellular methylation capacity and when it is decreased can correlate with reduced methylation potential (38, 39). An alteration of the methylation capacity is reported in DS and was connected to aging acceleration supporting the notion that DS is a progeroid trait (40).

Our statistical analyses report a negative correlation between SAM concentration levels and vitamin B12 (when the subjects are in a non-fasting state) (*r* = −0.628 and *p*-value = 0.029) and a positive correlation between SAM and 5-formyl-THF (*r* = 0.81 and p-value = 0.003) in plasma samples in the DS group (see Figure 3 and Supplementary Table 11). These findings are difficult to interpret but it is interesting to note that the association between SAM and 5-formyl-THF is present only in the DS group and is lost in the control group, suggesting again a selective alteration of one-carbon cycle in subjects with DS. Further studies are necessary to increase the number of SAM concentration values obtained from DS and control groups.

A point of strength of our analysis is the number of healthy controls that are siblings of children with DS included in the control group because the similarity in genetic background between siblings helps to highlight the differences due to the additional copy of Hsa21 in the trisomic subjects (an extreme of this condition has been found in literature with the use of cells derived from two monozygotic twins discordant for trisomy 21, (41). On the other hand, the similarity in the genetic background might prevent finding a difference when similarity in genetic polymorphisms related to the investigated pathway obscures a specific action of Hsa21 genetic determinants. To avoid this, we performed a reanalysis of the data following the removal of each metabolite data that was analyzed in both siblings and these analyses showed that almost identical results are obtained regarding differences for each metabolite between the two groups (Supplementary Tables 12, 13). Moreover, the mean DS/control ratio and the median DS/control ratio appear highly consistent with the same values already obtained when samples from pairs of DS and control subjects had been included in the analyses (Supplementary Table 13).

Abnormal folate metabolism has been causally linked with many diseases. Cerebral folate deficiency (CFD), caused by folate receptor autoantibodies or germline mutations such as in *FOLR1* (folate receptor alpha), *SLC46A1* (solute carrier family 46 member 1) or *MTHFR* genes, is manifested in neurological impairments and shows deficiency of 5-methyl-THF in the cerebrospinal fluid (CSF) in the presence of low or undefined peripheral folate levels (42, 43). Thus, folate metabolism plays a crucial role in the brain, but is still poorly defined.

Recently, we have explored the role of five metabolite levels involved in one-carbon pathway (Hcy, folate, vitamin B12, uric acid, creatinine) in the intellectual impairment of children with DS. The findings highlighted that specific vitamin B12 and Hcy thresholds corresponded respectively with better and worse cognitive scores in children with DS suggesting a role in their cognitive development (28).

Today the research about DS is focusing on the improvement of cognitive status (44). Several groups have tried to improve cognitive skills of subjects with DS using different compounds in clinical trials (45–49). Even if some of them showed interesting results, as of today none of these compounds are used as effective treatments of cognitive impairment in DS.

It was demonstrated that folate deficiencies contribute to several neuropsychiatric disorders and that can be corrected by folate supplementation (50–52). Three clinical trials tried to use 5-formyl-THF supplementation (alone and together with other compounds) to improve psychomotor and language development in young subjects with DS, but they did not evidence any significant clinical differences compared to placebo group (53–55). The search results for “Down syndrome” [MeSH Terms] AND “folic acid” [MeSH Terms] in Pubmed.gov^4^ did not retrieve any other clinical trial, about folates and DS.

Our results confirm that there is a dysregulation of the one-carbon pathway in subjects with DS that could be related to cognitive impairment. In particular, it is remarkable that plasma THF median concentration in subjects with DS appears to be lacking and exactly inversely proportional to the Hsa21 chromosomal dosage. This might imply a direct role of Hsa21 in impairing the progression of the folate/one-carbon cycle and opens the possibility that restoring a normal THF concentration could be important in subjects with DS. To this aim, we could evaluate administration of THF itself, or of the well known folinic acid, or of 5-methyl-THF.

Regarding THF, we have demonstrated that THF, as well as folic acid, is not able to rescue MTX toxicity in both euploid and T21 fibroblast cells, while 5-methyl-THF and 5-formyl-THF treatments have shown much better protective effects during MTX treatment (19). Moreover, the THF node of the one-carbon metabolic network is located far from the steps related to the alteration of the methylation capacity as highlighted by the SAM/SAH altered ratio already documented in DS and confirmed here as discussed above. Finally, it should be noted that to date THF supplement is not available for human administration.

Concerning folinic acid (5-formyl-THF), it has been in use for the longest time for the treatment of CFDs (56) and is currently the most used folate form for the treatment of CFDs (43). However, its inefficiency in any improvement of the cognitive skills in children with DS has already been clearly demonstrated (54, 55). This finding is again consistent with the position of this metabolite in the folate cycle in that it goes toward more oxidized forms of THF, while Hcy/methionine cycle requires more reduced ones.

The 5-methyl-THF is the most reduced folate form (57), the biologically active form of folate and it is essential for the formation of SAM, the universal methyl donor, by the regeneration of methionine from Hcy (see Figure 1). SAM is a powerful methylating agent in several biosynthetic reactions, and its methyl group is the most reactive in the one-carbon cycle (57). Moreover, and remarkably, 5-methyl-THF is the only folate form able to cross the blood-brain barrier (36). It is well absorbed in the intestinal tract and its bioavailability is not influenced by additional enzymatic steps (43). 5-methyl-THF has been made available for human administration only since 2000 (58) and it is still poorly used. One of the reasons for this is because it costs more than folic acid even though it is has been proposed to possibly be superior than folic acid in allowing the bypass of the pathway needed to generate 5-methyl-THF from folic acid, for example when the methylation step of THF is impaired in the carriers of the *MTHFR* C6777 polymorphism (59).

The use of 5-methyl-THF has more recently been proposed for the treatment of CFD (60, 61) and is considered to be the most efficient way to normalize CSF 5-methyl-THF concentrations (43). Although 5-methyl-THF plasma concentration is not decreased in the subjects with DS that we have investigated, from the above discussion it appears to be the best way to restore the THF deficit. In addition, considering that in subjects with CFD the peripheral blood 5-methyl-THF levels may be normal while they are low in the CSF (43), a similar condition might be present in DS. Even if a draw of CSF only for research appears unjustified to us, we plan to study 5-methyl-THF levels in samples available following neurosurgery in subjects with DS.

For all these reasons, we propose 5-methyl-THF as the best candidate for a clinical trial that aims to improve cognitive status of subjects with DS. Nevertheless, we advise against administrating 5-methyl-THF until the effective dosage able to correct the altered values is identified in a pilot study.

## Data availability statement

The datasets generated and analyzed during the current study have been made available as Supplementary Data Sheet 1. Further inquiries can be directed to the corresponding authors.

## Ethics statement

The studies involving human participants were reviewed and approved by Independent Ethics Committee of the Hospital - University of Bologna Policlinico S. Orsola-Malpighi, Italy (number: 39/2013/U/Tess). Written informed consent to participate in this study was provided by the participants’ legal guardian/next of kin.

## Author contributions

BV, PS, and FA designed the experiments. CL, GLP, and GC enrolled the subjects involved in the study. CG and GR collaborated in the enrollment of the subjects. BV, GZ, AnP, GLR, LV, GR, MCP, and FA collaborated in the treatment of the samples and performed ELISAs. BV, AnP, MC, AlP, PS, GR, and FA contributed to data analysis. GR, MCP, and FA supervised the project. All authors critically reviewed and approved the final manuscript.

## Footnotes

1
https://www.socscistatistics.com/tests/kolmogorov/default.aspx

2
https://www.graphpad.com/quickcalcs/ttest1.cfm

3
https://www.socscistatistics.com/tests/mannwhitney/

4
https://pubmed.ncbi.nlm.nih.gov/
