# Corrigendum: One-carbon pathway metabolites are altered in the plasma of subjects with Down syndrome: relation to chromosomal dosage

**DOI:** 10.3389/fmed.2024.1432559

**Published:** 2024-06-18

**Authors:** Beatrice Vione, Giuseppe Ramacieri, Giacomo Zavaroni, Angela Piano, Giorgia La Rocca, Maria Caracausi, Lorenza Vitale, Allison Piovesan, Caterina Gori, Gian Luca Pirazzoli, Pierluigi Strippoli, Guido Cocchi, Luigi Corvaglia, Chiara Locatelli, Maria Chiara Pelleri, Francesca Antonaros

**Affiliations:** ^1^Department of Experimental, Diagnostic and Specialty Medicine (DIMES), Unit of Histology, Embryology and Applied Biology, University of Bologna, Bologna, Italy; ^2^Department of Medical and Surgical Sciences (DIMEC), University of Bologna, Bologna, Italy; ^3^Medical Department, Maggiore Hospital, Bologna, Italy; ^4^Neonatology Unit, IRCCS Sant'Orsola-Malpighi University Hospital, Bologna, Italy

**Keywords:** trisomy 21, Down syndrome, one-carbon pathway, folates, chromosomal dosage

In the published article, as published in Table 1, the order of magnitude of the S-adenosyl-methionine (SAM) concentration was μg/mL, according to the ELISA kit specifications. After the publication of the manuscript, we conducted further analysis on the same type of samples using an ELISA kit of a different brand and a control chromatographic technique, suggesting that there was an error in the magnitude declared by the provider of the kit originally used in this paper and that the correct unit of measurement was ng/mL for SAM concentration. This has also been confirmed by the review of biomedical literature related to expected values of SAM in the human plasma (1). Based on the convergence of the literature review, the re-test by a different ELISA kit and a further independent test by liquid chromatography with tandem mass spectrometry (LC-MS/MS), which may be the better choice to analyze these small molecules (2), we believe that *bona fide* measurement unit for SAM has to be intended in our manuscript as ng/mL and not as μg/mL. Therefore, we have corrected this unit in the various sections of the paper to ensure the reproducibility of our data, while informing the reader about the discrepancy with the assay specifications provided with the kit, which is in any case no longer available for commercial reasons. The corrected Table 1 and its caption appear below.

**Table 1:** Main results of descriptive analysis of DS and control group excluding strong outliers.

	**DS (*****n*** = **164)**					**Control (*****n*** = **54)**				
	**THF (ng/mL)**	**5-methyl-THF (ng/mL)**	**5-formyl-THF (pg/mL)**	**SAH (ng/mL)**	**SAM (ng/mL)**	**THF (ng/mL)**	**5-methyl-THF (ng/mL)**	**5-formyl-THF (pg/mL)**	**SAH (ng/mL)**	**SAM (ng/mL)**
Valid *n* =	108	140	79	94	24	41	34	21	19	15
Missing n =	56	24	85	70	140	13	20	33	35	39
Mean	41.574	50.247	151.675	6.588	10.308	57.783	45.369	145.917	1.911	8.022
Median	33.955	49.766	137.955	6.298	9.514	51.471	42.744	139.144	1.736	5.222
SD	29.414	9.358	29.300	2.923	4.237	32.645	12.301	15.413	1.213	6.362

The values of subjects with Down syndrome (DS) are reported on the left of the table and the values of normal control subjects (control) are reported on the right of the table. For each metabolite the number (*n*) of plasma samples valid or missing are reported. Also the mean, median and standard deviation (SD) values are shown. The detailed results of descriptive analysis with and without strong outliers are shown in **Supplementary Table 6**.

The authors apologize for this error and state that this does not change the scientific conclusions of the article in any way, due to the fact that we reported and discussed results based on Down syndrome to normal ratio, and the ratio between the values remains unchanged following the change of measurement unit.

In the published article, there was an error in Figure 2 as published. For the same reasons reported above, the order of magnitude of the SAM concentration has been changed from μg/mL to ng/mL. The corrected Figure 2 and its caption appear below.

**Figure 2:**
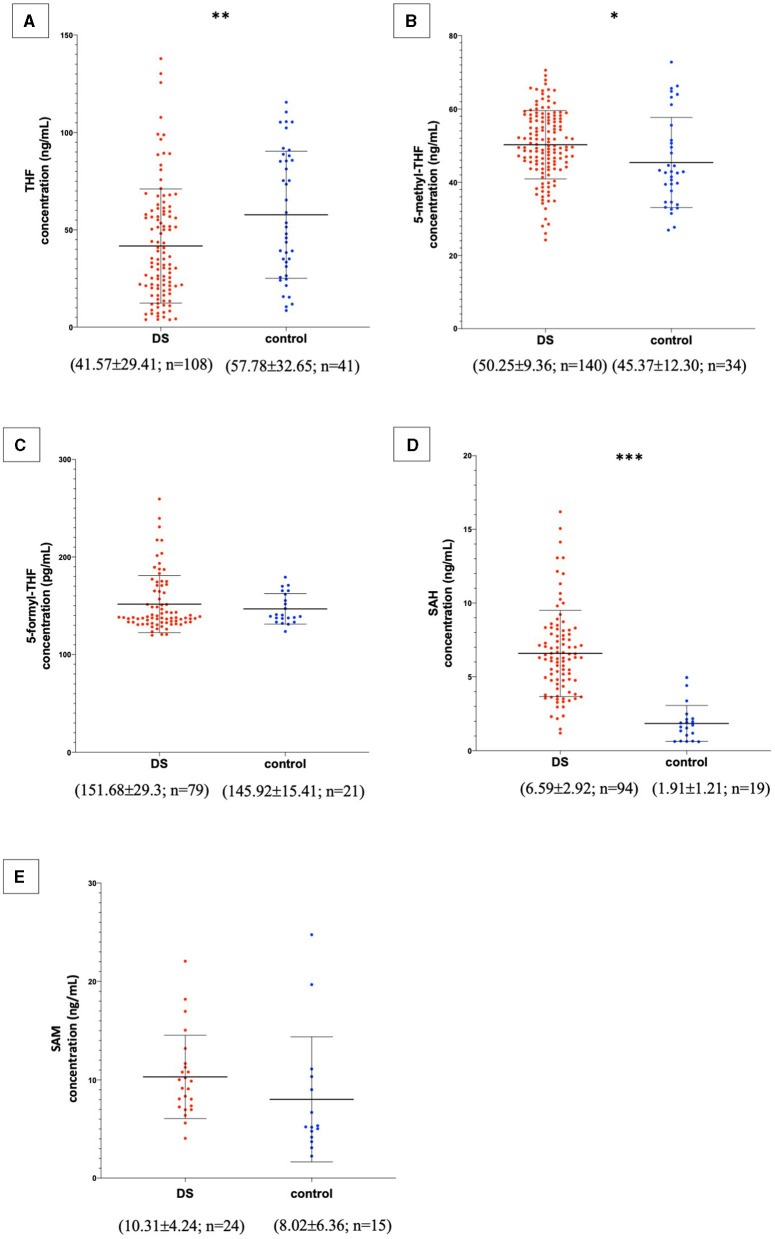
Metabolite concentrations in subjects with DS and normal control subjects. The graphs report metabolite plasma levels of each subject in the study. On the x-axis there is the subdivision of subjects in DS and control groups. Subjects with DS are represented like red dots and normal control subjects are represented like blue dots. On the *y*-axis the concentration of the metabolite in ng/mL or pg/mL is reported. The asterisks above the graph indicate the level of statistical significance (^*^p < 0.05; ^**^p < 0.005; ^***^p < 0.0005). The middle black lines indicate the mean concentration values for each group and the external black lines indicate standard deviation (SD) values. The mean concentration, SD values and the number of subjects (*n*) are reported below each graph for DS and control groups. **(A)** shows THF concentrations; **(B)** shows 5-methyl-THF concentrations; **(C)** shows 5-formyl-THF concentrations excluding strong outliers; **(D)** shows SAH concentrations excluding strong outliers; **(E)** shows SAM concentrations. The graphs were created with GraphPad Prism software v.6.0 (San Diego, CA).

In the published article, in the **Supplementary Tables 5, 6, 12**, the order of magnitude of the SAM concentration has been changed from μg/mL to ng/mL, for the same reasons reported above. The corrected Supplementary Tables have been uploaded in the appropriate section of the online version of the paper, replacing the previous files.

The authors apologize for these errors and state that this does not change the scientific conclusions of the article in any way. The original article has been updated.
