# Supplementary material for: One-carbon pathway metabolites are altered in the plasma of subjects with Down syndrome: Relation to chromosomal dosage
Source: Front Med (Lausanne). 2022 Dec 1;9:1006891. doi: 10.3389/fmed.2022.1006891 (PMC9751312; doi:10.3389/fmed.2022.1006891)
Supplement: Supplementary file 2 [file Image_2.PDF]

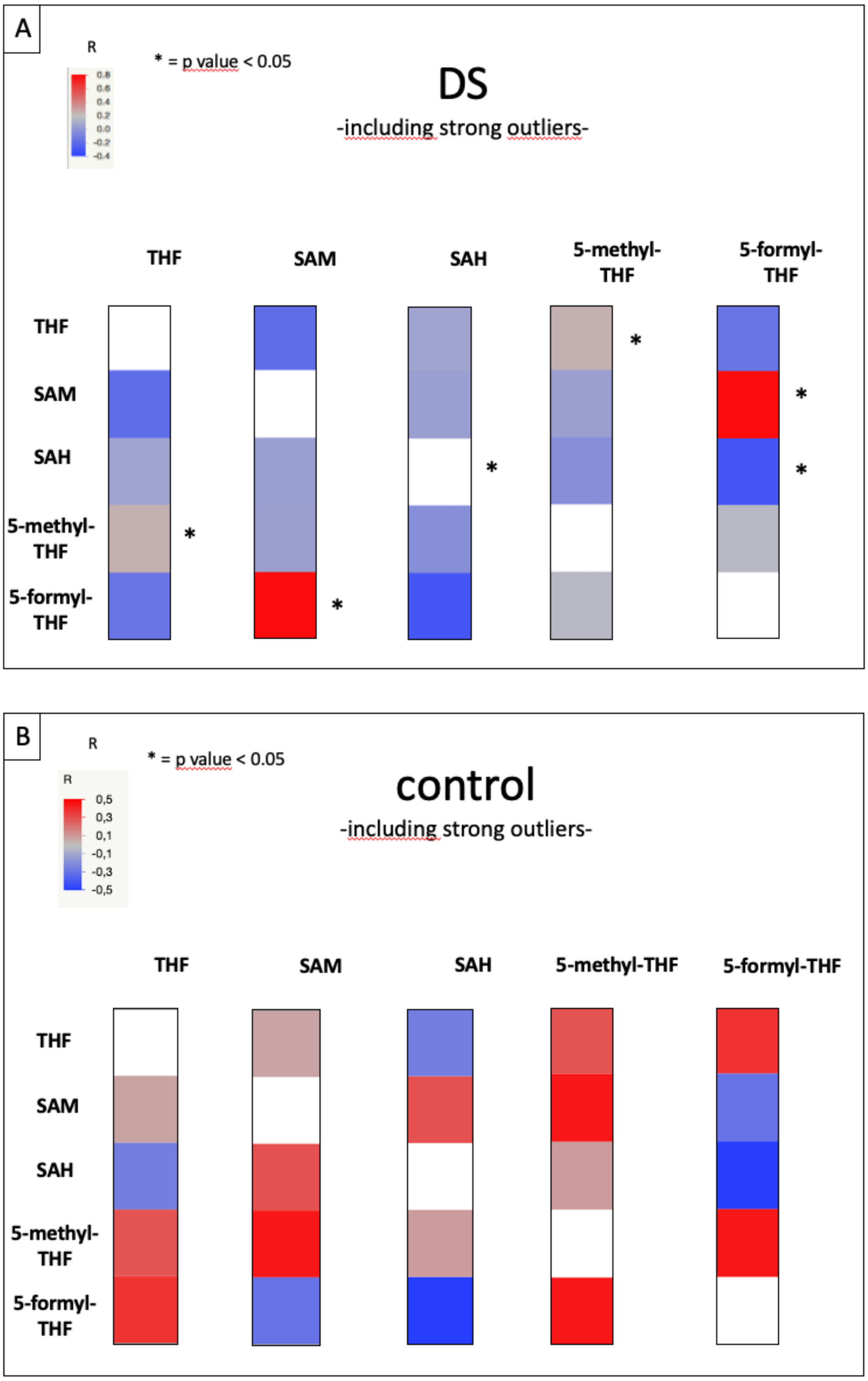

Supplementary Figure 2. Heat Map figure of bivariate correlation between levels of each metabolite and levels of all the other metabolites including strong outliers. Supplementary Figure 2A presents bivariate correlation in DS group and Supplementary Figure 2B presents bivariate correlation in control group (for complete data see Supplementary Table 11). At the top left of the figures the color code bar for Pearson correlation coefficient (r) is reported. Statistically significant correlations were marked with an *
