# Supplementary material for: One-carbon pathway metabolites are altered in the plasma of subjects with Down syndrome: Relation to chromosomal dosage
Source: Front Med (Lausanne). 2022 Dec 1;9:1006891. doi: 10.3389/fmed.2022.1006891 (PMC9751312; doi:10.3389/fmed.2022.1006891)
Supplement: Supplementary file 4 [file Data_Sheet_2.DOCX]

**Analysis performed in the paper**

**Descriptive analysis**

In order to perform descriptive analysis with SPSS Statistics software we did as follows: from the leading software Menu, we selected “Analyze” and then “Descriptive analysis”, we then chose “Frequencies”, we included our concentration data in the “variables” box and finally we chose “Mean”, “Median”, “Modal value”, “Standard deviation”, “Variance”, “Range”, “Minimum”, “Maximum” in the “Statistics” box.

The presence of strong outliers in concentration level distribution was performed with SPSS Statistics as follows: from the leading software Menu we selected “Analyze” and then “Descriptive statistics”, we then chose “Explore”, and included concentration levels in “Dependent List”, finally in “Statistics section” we selected the “Outliers” and “Percentiles” options. SPSS considers data as an outlier if it is outside the following ranges: above the 3^rd^ quartile +1.5 interquartile range or below the 1^st^ quartile -1.5 interquartile range and indicates it with an asterisk in the graph.

**Studying the distribution type:** **Kolmogorov-Smirnov test**

To check if our data followed a normal distribution we used Kolmogorov-Smirnov test using Social Science Statistic software online (<https://www.socscistatistics.com/tests/kolmogorov/default.aspx>) as follows: we included concentration levels in “Your data” tab and select “Calculate”.

**Comparing the mean of two groups: Unpaired Student t-test and Mann-Whitney U test**

For each metabolite (THF, 5-methyl-THF, SAH and SAM) we performed an unpaired student t-test between DS and control group using the “Graph Pad” t-test calculator online (<https://www.graphpad.com/quickcalcs/ttest1.cfm>) as follows: we chose “Enter or paste up to 2000 rows”, then we chose “Unpaired t-test”, entered for each DS metabolite data in “Group one” column and control data in “Group two” column and in the end, we chose “Calculate now”.

For 5-formyl-THF level we performed Mann Whitney test online (<https://www.socscistatistics.com/tests/mannwhitney/>) as follows: we included 5-f-THF levels of DS subjects in “Sample 1” tab and 5-f-THF levels of control subjects in “Sample 2”; we selected “Significance level at 0,05 “and “Two-tailed hypothesis” and select “Calculate U”.

Unpaired t-test was used to test whether sex and fasting/non-fasting state might affect the main results and it was performed with SPSS Statistics as follows: from the leading software Menu we selected “Analyze” and then “Compare means”, we then chose “independent-samples T-test” and finally we included our data in the “test variables” box and inserted “Sex” or “Fasting/non-fasting” in the “grouping variable” box.

**Building a Scatter Plot graph**

The graphic reports of each subject’s metabolite plasma levels in the study were created with GraphPad Prism software v.6.0 (San Diego, CA). To create a new graph of existing data: From the data table, we clicked the "Create New Graph" button then we clicked "Select" and choose the data sets to plot. We choosed "Column" graph, "Individual values", "Scatter plot" and "Mean with SD" in Plot box.

**Correlation analyses:**

**Linear correlation**

A linear correlation was used to determine if the correlation between age and molecule levels existed and it was performed with SPSS Statistics as follows: from the leading software Menu, we selected “Analyze” and then “Correlate”, we then chose “Bivariate correlation”, and finally we included our data in the “variables” box.

SPSS Statistics was used to perform a linear correlation between the level of each molecule and the levels of all the other molecules. Briefly, from the main Menu of the “SPSS Statistics” software, we selected “Analyze” and then “Correlation”; we chose “Bivariate” and finally we inserted our data in the main box.

**Partial correlation**

Partial correlation analyses checked for the effect of chronological age were used to investigate associations between the level of the involved molecules and other molecules. These analyses were performed as follows: from the main Menu of the “SPSS Statistics” software, we selected “Analyze” and then “Correlation”; we chose “Partial correlation” and finally we inserted our data in the main box and inserted “Age” in the “Check by” box.

**Building a Heat Map figure**

The Heat Map figures representing metabolite correlations (THF, 5-methyl-THF, 5-formyl-THF, SAH and SAM) were generated using JMP Pro software (Version 14 of the SAS System for Mac OS X, SAS Institute Inc., Cary, NC, USA) as follows: from the main menu we used “Graph builder”, then “Heatmap” in the options set above the Title, then we selected each metabolite level in X and Y axis.
